# Garbage codes as the underlying cause of death: a cross-sectional study, state of Rio de Janeiro, 1998–2023

**DOI:** 10.1590/S2237-96222026v35e20251137.en

**Published:** 2026-06-22

**Authors:** Luciana Freire de Carvalho

**Affiliations:** 1 Universidade Federal do Rio de Janeiro Instituto de Ciências Médicas Macaé RJ Brazil Universidade Federal do Rio de Janeiro, Instituto de Ciências Médicas, Macaé, RJ, Brazil; 2 Universidade Estácio de Sá Faculdade de Medicina Rio de Janeiro RJ Brazil Universidade Estácio de Sá, Faculdade de Medicina, Rio de Janeiro, RJ, Brazil

**Keywords:** Underlying Cause of Death, Vital Statistics, Data Accuracy, Health Information Systems, International Classification of Diseases, Causa Básica de Muerte, Estadísticas Vitales, Exactitud de los Datos, Sistemas de Información en Salud, Clasificación Internacional de Enfermedades

## Abstract

**Objectives:**

To describe the temporal evolution and geographic distribution of deaths due to garbage codes, as well as to analyze the prevalence and characteristics associated with the occurrence of garbage codes as the underlying cause of death in the state of Rio de Janeiro between 1998 and 2023.

**Methods:**

Cross-sectional study of deaths among residents of the state obtained from the Brazilian Mortality Information System (*Sistema de Informações sobre Mortalidade*, SIM). Garbage codes were classified according to the methodology of the Global Burden of Disease Study. Descriptive analyses of temporal evolution and distribution by municipalities were conducted. The associations with sociodemographic variables, place of occurrence, and health region were analyzed using Poisson regression with robust variance and described using prevalence ratios (PR) and their respective 95% confidence intervals (95%CI).

**Results:**

Of the 3,399,380 deaths, 1,510,135 (44.4%) were classified as garbage codes. The proportion remained high, ranging from 48.0% (1998) to 46.3% (2023), with a transient reduction to 38.1% in 2021. Level 1 (worst quality) accounted for the highest proportion (22.9%). Higher prevalences were associated with unknown sex (PR 1.18; 95%CI 1.11; 1.23), Black (PR 1.17; 95%CI 1.16; 1.17) and Indigenous race/skin color (PR 1.16; 95%CI 1.10; 1.22), age group ≥80 years (PR 1.74; 95%CI 1.71; 1.75), and deaths with unknown place of occurrence (PR 1.30; 95%CI 1.25; 1.34).

**Conclusion:**

Half of the deaths had the underlying cause recorded as a garbage code, with higher prevalence among unknown sex and place of occurrence, older adults, and Black and Indigenous race/skin color. Active investigation, algorithm-based redistribution, and database integration are promising for improving data quality.

## Resumen

**Objetivos:**

Describir la evolución temporal y la distribución geográfica de las defunciones por códigos basura, así como analizar la prevalencia y las características asociadas a la ocurrencia de códigos basura como causa básica de defunción en el estado de Río de Janeiro entre 1998 y 2023.

**Métodos:**

Estudio transversal de las defunciones de residentes del estado obtenidas del Sistema de Información sobre Mortalidad. Los códigos basura se clasificaron según la metodología del *Global Burden of Disease Study*. Se realizaron análisis descriptivos de la evolución temporal y de la distribución por municipios. La asociación con variables sociodemográficas, lugar de ocurrencia y región sanitaria se analizó mediante regresión de Poisson con varianza robusta, y se describió mediante la razón de prevalencia (RP) y sus respectivos intervalos de confianza del 95% (IC95%).

**Resultados:**

De las 3.399.380 defunciones, 1.510.135 (44,4%) fueron clasificadas como códigos basura”. La proporción se mantuvo elevada, variando de 48,0% (1998) a 46,3% (2023), con una reducción transitoria a 38,1% en 2021. El nivel 1 (de peor calidad) concentró la mayor proporción (22,9%). Las mayores prevalencias se asociaron con sexo ignorado (RP 1,18; IC95% 1,11; 1,23), raza/color de piel negra (RP 1,17; IC95% 1,16; 1,17) e indígena (RP 1,16; IC95% 1,10; 1,22), grupo de edad ≥80 años (RP 1,74; IC95% 1,71; 1,75) y defunciones en lugar ignorado (RP 1,30; IC95% 1,25; 1,34).

**Conclusión:**

La mitad de las defunciones presentó causa básica registrada como código basura, con mayor prevalencia en personas mayores, en población de raza/color de piel negra e indígena, y en personas de sexo masculino y lugar de ocurrencia ignorados. La investigación activa, la redistribución mediante algoritmos y la integración de bases de datos se presentan como estrategias prometedoras para mejorar la calidad de la información.

**Ethical aspects**

This research used public domain anonymized databases.

## Introducion

Mortality statistics are valuable sources of information for assessing population health status [1]. The usefulness of these data for public management depends on the correct identification of the underlying cause of death, defined as “the disease or injury that initiated the chain of pathological events leading to death or the circumstances of the accident or violence that produced the fatal injury” [2]. However, the quality of this information is often compromised by the use of garbage codes or ill-defined causes of death, which consist of undefined, incomplete, or insufficiently specific diagnoses that hinder the proper identification of priority public health actions [3].

To improve the response to this problem, the Global Burden of Disease Study (GBD) proposed a taxonomy that hierarchizes garbage codes into four levels, based on the degree of nonspecificity and the impact on the distortion of mortality statistics, with level 1 representing a very high impact and level 4 a low impact. This classification makes it possible to identify causes that prevent recognition of the disease group, as well as diagnoses that lack further detail. The adoption of this typology supports processes of death redistribution and guides managers in prioritizing interventions [4].

Although cause-specific mortality statistics are important for characterizing the epidemiological profile of the population and for prioritizing health actions and policies [5], the low quality of databases limits such analyses, especially in developing countries [5–7]. A high proportion of garbage codes compromises the quality of information on causes of death. It has been used as an indicator for assessing quality of care, since inequalities in access to and quality of medical care are related to the frequency of deaths from ill-defined causes [7,8]. It is estimated that when the proportion of garbage codes exceeds 10% of total deaths, mortality data become compromised, making it difficult to obtain reliable statistics [9].

In Brazil, proportions of garbage codes above 30% persist [3,9–11]. Yet, the literature on the subject still lacks localized studies, which justifies this analysis’s contribution to the territory of the state of Rio de Janeiro.

The objectives of this study were to describe the temporal evolution and geographic distribution of deaths due to garbage codes, and to analyze the prevalence and characteristics associated with garbage codes as the underlying cause of death in the state of Rio de Janeiro between 1998 and 2023.

## Methods

### Study design, setting, period, and population

This is a cross-sectional study based on individual-level data from all deaths of residents in the state of Rio de Janeiro recorded in the Brazilian Mortality Information System (*Sistema de Informação sobre Mortalidade*, SIM) between January 1, 1998, and December 31, 2023. This state comprises 92 municipalities distributed across nine health regions, with an estimated population of 16,054,524 inhabitants in 2022 [12].

Descriptive analyses aggregated by underlying cause, year, and municipality were conducted to characterize the spatial and temporal context of deaths, and individual-level analyses were conducted to estimate prevalence and identify factors associated with the recording of garbage codes. 

### Variables 

The classification of the dependent variable in this study was defined by the presence of any underlying cause of death classified as a garbage code at levels 1–4, following the GBD methodology. The underlying cause of death was coded according to the International Statistical Classification of Diseases and Related Health Problems, 10th Revision (ICD-10), using four-digit codes.

The methodology followed the classification of garbage codes proposed by the GBD 2017, which categorized ICD-10 codes into four levels of quality (Supplementary Table 1): 

**Table 1:** Five most frequent underlying causes of death at each level of garbage codes, according to the Global Burden of Disease Study (GBD), with absolute frequencies and proportions for each level. State of Rio de Janeiro, 1998–2023 (n=1,510,135)

ICD-10 code^a^	Description of the underlying cause	n (%)^b^
**Level 1 **– **Worst quality**		
R99	Other ill-defined and unspecified causes of mortality	232,787 (32.5)
E149	Unspecified Diabetes mellitus	87,567 (12.2)
A419	Unspecified septicemia	77,872 (10.9)
I500	Congestive heart failure	36,632 (5.1)
I509	Unspecified heart failure	27,350 (3.8)
**Level 2 **– **Low quality**		
I10	Essential (primary) hypertension	88,799 (39.4)
Y349	Events of undetermined intent	31,023 (13.8)
R092	Respiratory arrest	19,104 (8.5)
J81	Pulmonary edema	14,281 (6.3)
K920	Hematemesis	11,862 (5.3)
**Level 3 **– **Intermediate quality**		
J984	Other pulmonary disorders	20,360 (17.4)
C80	Malignant neoplasm without specification of site	16,706 (14.3)
I499	Unspecified cardiac arrhythmia	9,653 (8.2)
J988	Other specified respiratory disorders	9,245 (7.9)
C260	Malignant neoplasm of unspecified part of the intestinal tract	7,928 (6.8)
**Level 4 **– **Best quality**		
J189	Unspecified pneumonia	150,091 (33.2)
I64	Stroke, not specified as hemorrhagic or ischemic	120,713 (26.7)
I678	Other specified cerebrovascular diseases	47,548 (10.5)
I694	Sequelae of stroke, not specified as hemorrhagic or ischemic	28,541 (6.3)
I420	Dilated cardiomyopathy	24,126 (5.3)

^a^International Classification of Diseases – 10th Revision (ICD-10); ^b^Proportions were calculated based on the total number of deaths in each GBD level, considering only the five most frequent causes per level.

- level 1 (worst quality) – includes codes with serious implications for public policy, such as septicemia, which can be redistributed to any major group;
- level 2 (low quality) – includes codes with substantial implications for causes that can be redistributed into one or two major groups, such as essential hypertension;
- level 3 (intermediate quality) – includes codes with important implications for causes that are likely within the same ICD-10 chapter, such as cancer without specification of site; and
- level 4 (best quality) – includes causes that refer to a single disease but could be further specified, such as stroke not specified.

These codes are subsequently reassigned to probable underlying causes using redistribution algorithms based on proportions of high-quality records from the same population (15).

The independent variables selected for the analysis were: sex (male, female, unknown), race/skin color (White, Black, Yellow, Brown [Brazilian mixed race], Indigenous, Unknown), age group (<1, 1–9, 10–19, 20–39, 40–59, 60–79, and ≥80 years), place of occurrence of death (hospital, home, public road, other health facilities, other, unknown), health region (*Baía da Ilha Grande, Baixada Litorânea, Centro-Sul, Médio Paraíba, Metropolitana I, Metropolitana II, Noroeste, Norte, and Serrana*), and year of death (1998–2023).

Years of death were analyzed as continuous (1998–2023) in the descriptive analysis and as categorical in the multivariable analysis. For presentation in the regression table, the following were selected: 1998 (beginning of the historical series with ICD-10 showing stability); 2011 (midpoint of the series, with intermediate quality after national investments in surveillance); 2021 (most critical moment of the COVID-19 pandemic); and 2023 (most recent available year).

### Data sources and measurement

The data source was the SIM, made available by the Brazilian Unified Health System Information Technology Department (*Departamento de Informática do Sistema Único de Saúde*, DATASUS), whose database with unidentified information is publicly available in the OpenDATASUS repository [13].

The processing of mortality data in the state of Rio de Janeiro follows a decentralized flow. The cycle begins with physicians in reporting units issuing death certificates, with municipal health departments responsible for data collection, entry, and processing. The coding of causes and the selection of the underlying cause are performed by trained coders linked to municipal and state levels, with the aid of the Underlying Cause Selection software to apply ICD-10 rules. The state level monitors quality, corrects inconsistencies, and validates the data before final processing by DATASUS [14].

For the analysis, the original database provided by the Ministry of Health was used, without excluding records due to incomplete sociodemographic variables. Categories classified as “unknown” were maintained and analyzed as such, reflecting the real scenario of death certificate completion during the period. 

### Statistical methods 

Initially, descriptive analyses were performed of the distribution of the total number of deaths (n) and the proportions (%) of garbage codes for each category of the study variables. The proportion of total garbage codes and by levels over the years was presented in a graph.

The geographic distribution of the proportion of garbage codes by municipality was visualized in maps, using the municipality as the unit of analysis and the equal interval classification method, with the definition of five fixed ranges of 10 percentage points each (20%–30%, 31%–40%, 41%–50%, 51%–60%, and >60%). Additionally, the results were stratified by the state’s nine health regions, allowing comparison of information quality performance across these regional aggregates.

A ranking of the five most frequent underlying causes of death was performed for each of the four quality levels proposed by the GBD, using absolute frequency (N) and the proportion of each cause within its level. Proportions were calculated using the total number of deaths classified at each GBD level as the denominator, and the five most frequent causes were presented for each level.

Due to the high prevalence of the outcome, the association between dependent and independent variables was modeled using Poisson regression with robust variance, an appropriate approach for obtaining an adjusted prevalence ratio (PR) in cross-sectional studies with common outcomes, avoiding overestimation of the magnitude of association resulting from the use of the odds ratio (OR).

Associations were initially investigated through univariate analyses to obtain unadjusted PR. Subsequently, multivariable modeling was performed, in which the final model was adjusted simultaneously for sex, race/skin color, age group, place of occurrence, health region, and year of death. This approach was adopted because it is a long historical series in which adjustments are necessary to control for confounding factors arising from changes in the demographic and spatial composition of the population. This ensures that the PR reflects associations adjusted for each variable analyzed.

Estimates of association were expressed as PR, accompanied by their respective 95% confidence intervals (95%CI). Statistical significance was assessed using the Wald test with robust standard errors, at a 5% significance level.

Analyses were performed using Stata 14.2 and R Studio 4.0.2.

## Results

In the period 1998–2023, 3,399,380 deaths of residents in the state of Rio de Janeiro were analyzed, of which 1,510,135 (44.4%) were classified as garbage codes.

The proportion of deaths due to garbage codes in the state remained high over the years (Figure 1A), ranging from 48.0% in 1998 to 46.3% in 2023. The lowest proportions were observed in 2021 (38.1%) and 2020 (38.8%), while the highest were in 1998 (48.0%) and 2019 (47.4%). 

**Figure 1:**
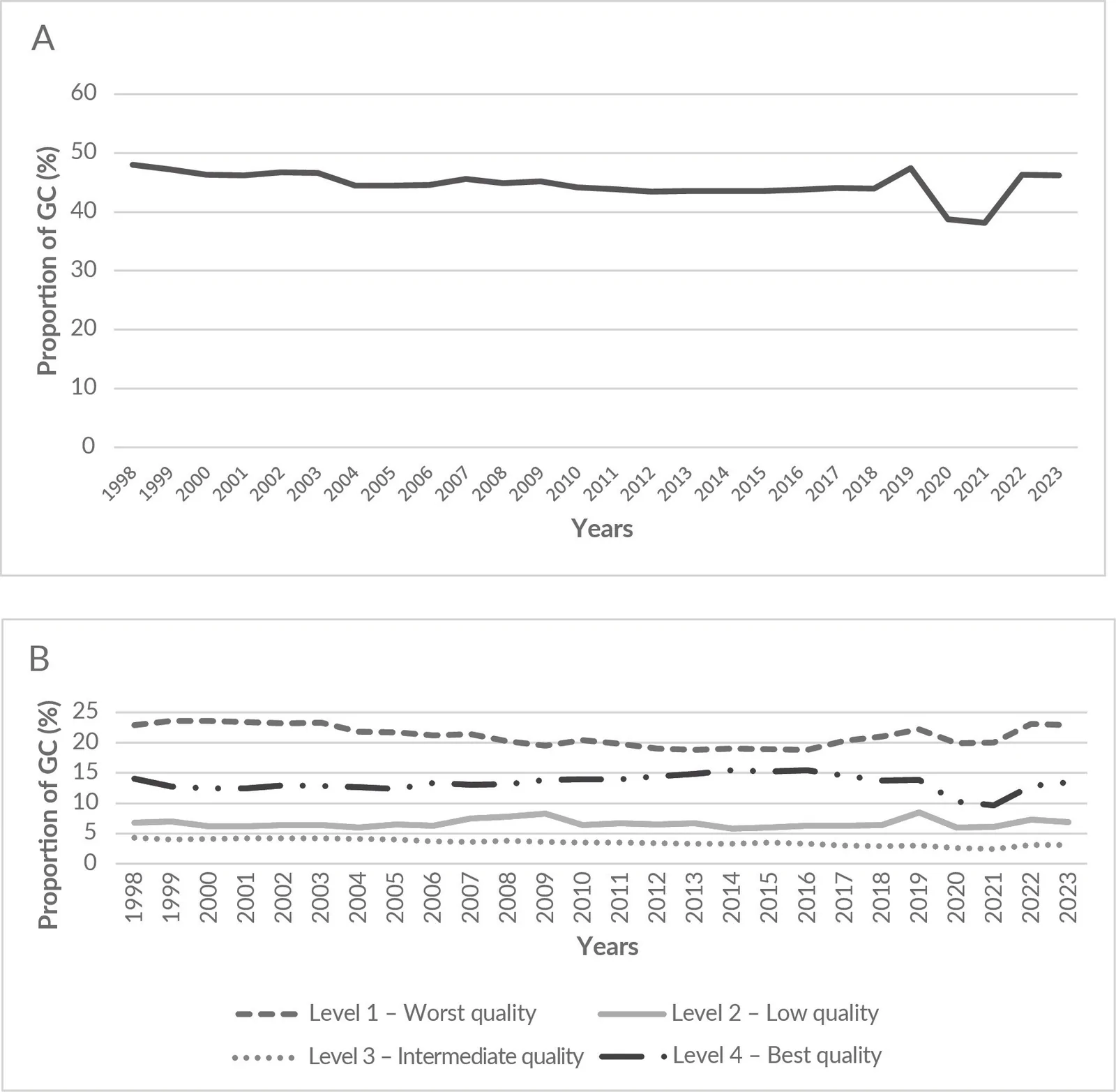
Temporal trend (A) and distribution by levels (B) of the proportion (%) of deaths due to garbage codes (GC). State of Rio de Janeiro, 1998–2023 (n=1,510,135)

The proportion of deaths attributable to garbage codes by GBD quality level, relative to total deaths, was detailed (Figure 1B). Level 1 encompassed the most nonspecific and lowest-quality causes. This level accounted for the highest proportion, remaining at 22.9% at the beginning (1998) and at the end of the series (2023), with its lowest point in 2016 (18.8%). This was followed by level 4, with variation from 14.0% in 1998 to 13.4% in 2023, with a minimum of 9.6% in 2021. Level 2 varied from 6.8% in 1998 to 7.3% in 2023, and level 3 from 4.3% in 1998 to 3.1% in 2023, reaching its minimum of 2.4% in 2021. A decline in the proportions of garbage codes was observed across all levels in 2020 and 2021. This decline was followed by a substantial increase in 2022 and 2023, with proportions returning to levels close to the pre–COVID-19 pandemic period.

A ranking of the five most frequent causes of garbage code at each GBD quality level was presented (Table 1). At level 1, cause R99 (other ill-defined and unspecified causes of death) was the most frequent and corresponded to 32.5% of deaths at this level (n=232,787). At level 2, essential (primary) hypertension (I10) accounted for the highest proportion of deaths, 39.4% (n=88,799). Level 3 highlighted other pulmonary disorders (J98.4, 17.4%) and malignant neoplasm without specification of site (C80, 14.3%). At level 4, unspecified pneumonia (J18.9, 33.2%) and unspecified stroke (I64, 26.7%) were the most common causes.

The geographic distribution of the proportion of deaths due to garbage codes by municipality was illustrated for 1998, 2011, 2021, and 2023 (Figure 2). In 1998 (Figure 2A), the highest proportions were observed in municipalities in the Noroeste Region of Rio de Janeiro, such as Varre-Sai (68.4%), and in the *Metropolitana I* Region, in Queimados (68.1%). General variation toward darker tones (higher proportions) was observed across much of the state. 

**Figure 2:**
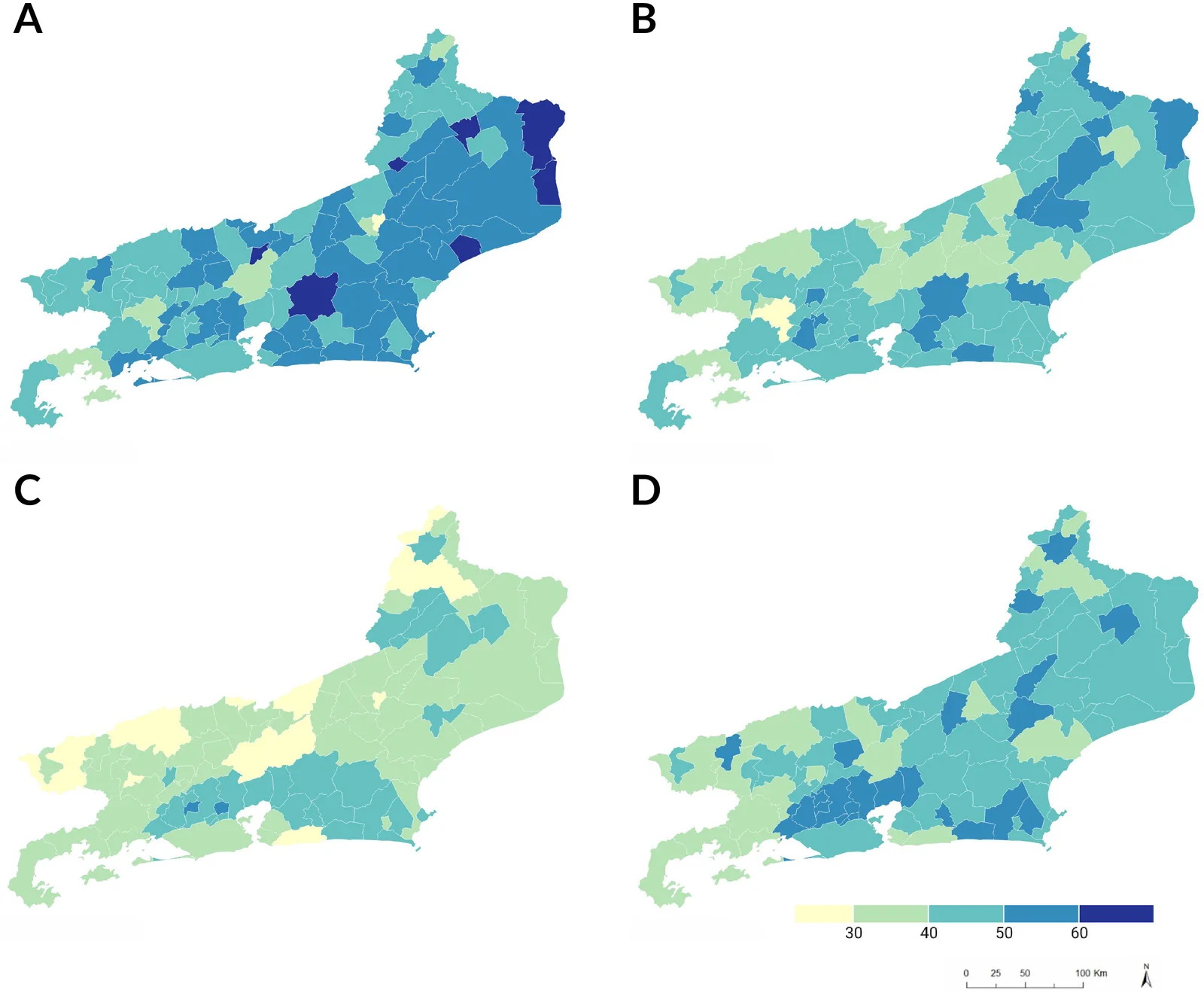
Proportion of deaths due to garbage codes by municipality: (A) 1998, (B) 2011, (C) 2021, and (D) 2023. State of Rio de Janeiro (n=1,510,135)

In 2011 (Figure 2B), most municipalities showed a reduction in the proportion of deaths due to garbage codes, as indicated by the lightening of the maps. Regions such as *Metropolitana I*, *Metropolitana II*, and *Centro-Sul *exhibited the lowest proportions, with Areal (34.44%) in Centro-Sul and Niterói (37.5%) in Metropolitana II among the municipalities with the lowest proportions. Municipalities in the Noroeste Region, such as Varre-Sai (47.5%), still accounted for a high proportion. 

In 2021 (Figure 2C), the proportion of deaths due to garbage codes declined across much of the state compared to previous years, reflecting the COVID-19 pandemic period. Municipalities in the Serrana Region, such as Petrópolis (27.9%), *São José do* *Vale do Rio Preto* (29.5%), and *São Sebastião do Alto* (38.0%), presented the lowest proportions. 

In 2023 (Figure 2D), the proportion of deaths due to garbage codes increased substantially in several regions, with the map darkening again compared to 2021. The highest proportions were recorded in municipalities in the *Baixada Litorânea* Region, such as *Rio das Ostras* (59.48%), and in the Metropolitana I Region, such as Belford Roxo (50.3%). 

Adjusted PR for the proportion of deaths due to garbage codes were presented, along with their respective 95%CI (Table 2). All categories showed statistical significance (p-value<0.001). The prevalence of deaths due to garbage codes was 6.0% higher in females compared to males (PR 1.06; 95%CI 1.06; 1.06). For race/skin color, the highest adjusted PR were observed in Black (PR 1.17; 95%CI 1.16; 1.17) and Indigenous populations (PR 1.16; 95%CI 1.10; 1.22) compared to the White population. 

**Table 2:** Unadjusted and adjusted prevalence ratios (PR) and 95% confidence intervals (95%CI) for deaths due to garbage codes by sociodemographic characteristics. State of Rio de Janeiro, 1998–2023 (n=3,399,380)

Variables	Total deaths	Deaths classified as garbage codes – n (%)	Unadjusted PR (95%CI)	p-value	Adjusted PR (95%CI)^a,d^	p-value
Sex				<0.001		<0.001
Male	1,837,839	770,190 (41.9)	1		1	
Female	1,558,618	738,311 (47.4)	1.13 (1.12; 1.13)		1.06 (1.06; 1.06)	
Unknown	2,923	1,634 (55.9)	1.33 (1.29; 1.38)		1.18 (1.11; 1.23)	
**Race/skin color**				<0.001		<0.001
White	1,827,802	789,877 (43.2)	1		1	
Black	489,258	233,242 (47.7)	1.10 (1.10; 1.11)		1.17 (1.16; 1.17)	
Asian	5,822	2,671 (45.8)	1.06 (1.03; 1.09)		1.06 (1.03; 1.09)	
Brown [Brazilian mixed race]	961,009	428,903 (44.6)	1.03 (1.03; 1.04)		1.13 (1.12; 1.13)	
Indigenous	1,713	834 (48.7)	1.13 (1.07; 1.18)		1.16 (1.10; 1.22)	
Unknown	113,776	54,608 (48.0)	1.11 (1.10; 1.12)		1.15 (1.13; 1.15)	
**Age group **(years)^b^				<0.001		<0.001
<1	88,298	14,671 (16.6)	0.56 (0.55; 0.57)		0.52 (0.51; 0.53)	
1–9	21,756	9,005 (41.4)	1.40 (1.37; 1.43)		1.30 (1.27; 1.32)	
10–19	60,090	17,799 (29.6)	1		1	
20–39	292,051	91,843 (31.4)	1.06 (1.05; 1.08)		1.04 (1.02; 1.05)	
40–59	679,181	272,006 (40.0)	1.35 (1.34; 1.37)		1.28 (1.26; 1.29)	
60–79	1,341,140	617,182 (46.0)	1.55 (1.53; 1.57)		1.49 (1.46; 1.50)	
≥80	916,864	487,629 (53.2)	1.80 (1.77; 1.82)		1.74 (1.71; 1.75)	
**Place of occurrence**				<0.001		<0.001
Hospital	2,441,787	1,062,323 (43.5)	1		1	
Unknown	3,269	1,759 (53.8)	1.24 (1.20; 1.28)		1.30 (1.25; 1.34)	
Other health facilities	255,956	139,001 (54.3)	1.25 (1.24; 1.25)		1.26 (1.25; 1.26)	
At home	468,159	227,807 (48.7)	1.12 (1.11; 1.12)		1.08 (1.07; 1.08)	
Public road	129,966	37,181 (28.6)	0.66 (0.65; 0.66)		0.74 (0.72; 0.74)	
Other	100,243	42,064 (42.0)	0.96 (0.96; 0.97)		1.01 (1.00; 1.02)	
**Health region**				<0.001		<0.001
Baía da Ilha Grande	36,804	14,269 (38.8)	0.86 (0.85; 0.87)		0.92 (0.90; 0.92)	
Baixada Litorânea	137,815	62,673 (45.5)	1.00 (1.00; 1.01)		1.04 (1.03; 1.04)	
Centro-Sul	70,304	30,957 (44.0)	0.97 (0.96; 0.98)		0.98 (0.97; 0.99)	
Médio Paraíba	168,556	66,614 (39.5)	0.87 (0.87; 0.88)		0.90 (0.89; 0.90)	
Metropolitana I	2,111,347	955,390 (45.2)	1		1	
Metropolitana II	403,987	175,141 (43.3)	0.96 (0.95; 0.96)		0.97 (0.97; 0.98)	
Noroeste	72,405	31,117 (43.0)	0.95 (0.94; 0.96)		0.95 (0.94; 0.96)	
Norte	159,752	72,305 (45.3)	1.00 (0.99; 1.01)		1.04 (1.03; 1.04)	
Serrana	195,953	79,614 (40.6)	0.90 (0.89; 0.90)		0.92 (0.91; 0.92)	
Unknown	42,457	22,055 (51.9)	1.15 (1.14; 1.16)		1.08 (1.07; 1.09)	
**Year of ^deathc^**^bold^>				0.001		0.001
1998	115,382	55,383 (48.1)	1		1	
2011	127,095	55,718 (43.8)	0.91 (0.90; 0.92)		0.85 (0.84; 0.86)	
2021	189,232	72,165 (38.1)	0.79 (0.79; 0.80)		0.71 (0.70; 0.71)	
2023	145,148	67,133 (46.3)	0.96 (0.96; 0.97)		0.85 (0.84; 0.86)	

^a^Adjusted for all independent variables; ^b^The 10–19 age group was selected as the reference category because it presented the second lowest prevalence of garbage codes, immediately after those under 1 year of age, whose deaths are legally required to be investigated; ^c^Death years were analyzed without temporal stratification; however, for presentation, specific years were selected corresponding to: 1998 (beginning of the historical series with stable ICD-10 implementation); 2011 (midpoint of the study period, with intermediate quality following national investments in surveillance); 2021 (critical moment of the COVID-19 pandemic); and 2023 (most recent available year, prioritizing visual clarity at key points); ^d^The overlap of the 95%CI limits with the point estimate is due to the high statistical precision resulting from the large sample size, while maintaining standardization to two decimal places.

The prevalence of deaths due to garbage codes was lower among children under 1 year of age (PR 0.52; 95%CI 0.51; 0.53) and increased with age, being 74.0% higher for those aged 80 years or older compared to the 10–19 age group (PR 1.74; 95%CI 1.71; 1.75). 

Prevalence was higher in the categories unknown (PR 1.30; 95%CI 1.25; 1.34) and other health facilities (PR 1.26; 95%CI 1.25; 1.26) compared to deaths occurring in hospitals, and was 26.0% lower for deaths on public roads (PR 0.74; 95%CI 0.72; 0.74). The health regions of *Baixada Litorânea* (PR 1.04; 95%CI 1.03; 1.04) and Norte (PR 1.04; 95%CI 1.03; 1.04) presented higher PR than Metropolitana I, whereas *Médio Paraíba* (PR 0.90; 95%CI 0.89; 0.90), Serrana (PR 0.92; 95%CI 0.91; 0.92), and Baía da Ilha Grande (PR 0.92; 95%CI 0.90; 0.92) had the lowest. 

A reduction in the prevalence of deaths due to garbage codes was observed over the time series. Compared with 1998, the PR was lower in all subsequent years evaluated, reaching its lowest point in 2021 (PR 0.71; 95%CI 0.70; 0.71). In 2023, the adjusted prevalence remained 15.0% below that observed in 1998 (PR 0.85; 95%CI 0.84; 0.86).

## Discussion

The results of this study stood out for the magnitude and persistence of deaths classified as garbage codes, indicating historical challenges in improving the quality of mortality information in the state of Rio de Janeiro. Despite advances, their occurrence remained consistently above levels considered acceptable to ensure reliable mortality statistics [9], and this was corroborated by findings in other Brazilian territories, which showed that 19 states had a proportion of garbage codes above 30% in 2016 and none below 10% [3,6,9,11,15]. 

The reduction in the proportion of garbage codes between 2020 and 2021 may be attributed to stricter death investigation procedures, the specificity of COVID-19 as a well-defined underlying cause of death, and the mobilization of surveillance systems in the state of Rio de Janeiro. This hypothesis differs from other studies conducted during the pandemic, which observed an increase in this period. However, caution is warranted when interpreting this reduction as a structural improvement in quality, since prioritization may have occurred in selecting COVID-19 as the cause at the expense of underlying chronic conditions. [10,16,17].

The subsequent increase in the proportions of garbage codes in 2022 and 2023 suggested that the improvement in data quality was not sustained, with levels of non-specificity returning to high levels in the post–COVID-19 pandemic period.

The concentration of garbage codes at level 1 represented the most severe impairment in information accuracy. The predominance of codes such as R99 (other ill-defined causes) limited the use of statistics, as the complete absence of etiological detail made it impossible to identify the true cause of death and to define priorities for public health planning. This reinforces the need for investments in death investigation and improved training of professionals responsible for completing death certificates [4,18]. 

Codes classified in levels 2 and 3, such as essential hypertension and malignant neoplasm without defined anatomical location, respectively, had the potential for greater refinement than level 1 codes, as they at least clarified the organ system or group of causes involved. However, they still represented a missed opportunity to deepen knowledge about morbidity and mortality due to noncommunicable chronic diseases in the state of Rio de Janeiro [19,20].

Disparities in the proportion of garbage codes across health regions and municipalities reflected the heterogeneity of health conditions and recording capacity in the state. These findings indicated possible inequalities in the accuracy of information, local capacity for death investigation, and, ultimately, in the provision of and access to health services [5].

The results reinforced the role of garbage codes as an indirect indicator of weaknesses in health care, especially in inland municipalities or in vulnerable metropolitan areas, where high concentrations may signal problems in coverage, quality of care, and technical training [16]. Municipalities with lower proportions of garbage codes may indicate better local practices in the completion or investigation of deaths. In contrast, those with higher proportions indicate the need for targeted interventions [21], especially when prevalence exceeds 50% over 25 years.

The differences observed in the prevalence of garbage codes stratified by sociodemographic variables underscored the influence of social determinants of health and inequities in access to and quality of care among population subgroups [7]. The highest PR associated with Black and Indigenous race/skin color reinforces the correlation between information quality and health inequities. This suggests barriers to access to diagnostic investigation, which may extend to the death certification process, resulting in less accurate data [22].

Regarding sex, a higher prevalence was observed in female deaths. This may be associated with the higher occurrence of deaths from external causes among men, which are generally certified by forensic physicians after autopsies, leading to a more specific diagnosis of causes of death for males [17].

The lower PR among children under 1 year of age suggested greater rigor in the investigation of infant deaths. In contrast, the progressive increase in older age groups pointed to diagnostic complexity and the prevalence of comorbidities in older adults as factors contributing to the nonspecificity of causes of death in this group. This fact could also explain the higher frequency among women, given their longer life expectancy than men [17,23].

The association of higher prevalences of garbage codes in deaths occurring at home, on public roads, or in other health facilities, compared to hospital deaths, is a pattern consistent with the literature. Deaths outside the hospital setting generally lack more detailed clinical information, making it more challenging to determine the underlying cause. Conversely, the lower proportion on public roads may be related to better investigation of deaths from external causes, since these are legally required to be investigated by forensic physicians through autopsies, which results in specific underlying causes [3,8,9]. 

Although the proportion of garbage codes remained high over time, the PR analysis revealed a trajectory of gradual improvement in information quality across the historical series. This decreasing trend, although insufficient, suggested that investments in surveillance and in improving the completion of death certificates may have had a positive effect over the years. 

The findings reinforced the need for continued investment in training health professionals who certify and investigate causes of death, especially physicians and surveillance teams. The urgency of strengthening the death surveillance network in the state was highlighted, with special attention to municipalities with higher proportions of ill-defined causes of death. 

Death investigation is an essential strategy for improving the quality of information and should be integrated into the workflows of local surveillance teams. By actively investigating deaths of nonspecific cause, surveillance services correct data distortions and identify weaknesses in care and barriers to access, enabling targeted interventions for territories and population groups most affected. 

Another strategy is to reclassify deaths coded as garbage codes using standardized redistribution algorithms. Studies applying this correction have shown that the mortality profile is substantially altered, revealing the true burden of specific causes [20,28,29]. 

The integration of information systems through data linkage techniques serves as a tool to improve the investigation of deaths from ill-defined causes. The relationship between SIM records and care bases enables the recovery of care trajectories and clinical data that are absent from the death certificate. This strategy, by enriching the context of death, can support the reclassification of these codes into specific causes, providing greater reliability to mortality statistics [30]. 

Among the limitations, the use of secondary data from the SIM is subject to information bias related to underreporting, as well as incompleteness and inconsistencies in the completion of death certificates. Although the GBD methodology is widely accepted, it may not translate the particularities of the Brazilian context. As this is a cross-sectional study, the associations did not allow causal inferences. The presence of unknown information may have influenced the estimates of adjusted PR. However, the large volume of data and the use of a validated methodology strengthened the robustness of the results.

In conclusion, the state of Rio de Janeiro faces challenges in the quality of mortality data, characterized by a high magnitude and persistence of garbage codes, with marked temporal and territorial heterogeneity associated with sociodemographic inequalities. The results of this study enable prioritization of the most vulnerable municipalities, regions, and subgroups to implement continuous strategies to improve the completion of death certificates and the investigation of causes of death, thereby strengthening vital statistics for the planning and evaluation of health policies.

## Data Availability

Derived datasets and analysis codes are available upon request from the author.
