# Supplementary material for: Garbage codes as the underlying cause of death: a cross-sectional study, state of Rio de Janeiro, 1998–2023
Source: Epidemiol Serv Saude. 2026 Jun 22;35:e20251137. doi: 10.1590/S2237-96222026v35e20251137.en (PMC13686309; doi:10.1590/S2237-96222026v35e20251137.en)
Supplement: Translation

# Translation: Códigos garbage como causa básica de óbito: estudo transversal, estado do Rio de Janeiro, 1998-2023

**DOI:** 10.1590/S2237-96222026v35e20251137.pt

**Authors:** Luciana Freire de Carvalho

**Affiliations:** 1 Universidade Federal do Rio de Janeiro Instituto de Ciências Médicas Macaé RJ Brasil Universidade Federal do Rio de Janeiro, Instituto de Ciências Médicas, Macaé, RJ, Brasil; 2 Universidade Estácio de Sá Faculdade de Medicina Rio de Janeiro RJ Brasil Universidade Estácio de Sá, Faculdade de Medicina, Rio de Janeiro, RJ, Brasil

**Keywords:** Causa Básica de Morte, Estatísticas Vitais, Qualidade dos Dados, Sistemas de Informação em Saúde, Classificação Internacional de Doenças

## Abstract

**Objetivos:**

Descrever evolução temporal e distribuição geográfica dos óbitos por códigos garbage, além de analisar a prevalência e as características associadas à ocorrência de códigos garbage como causa básica de óbito no estado do Rio de Janeiro entre 1998 e 2023.

**Métodos:**

Estudo transversal dos óbitos de residentes do estado obtidos do Sistema de Informações sobre Mortalidade. Os códigos garbage foram classificados segundo metodologia do Global Burden of Disease Study. Realizaram-se análises descritivas da evolução temporal e distribuição por municípios. A associação com variáveis sociodemográficas, local de ocorrência e região de saúde foi analisada por regressão de Poisson com variância robusta e descrita a partir da razão de prevalência (RP) e dos respectivos intervalos de confiança de 95% (IC95%).

**Resultados:**

Dos 3.399.380 óbitos, 1.510.135 (44,4%) foram classificados como códigos garbage. A proporção permaneceu elevada, variando de 48,0% (1998) a 46,3% (2023), com redução transitória para 38,1% em 2021. O nível 1 (pior qualidade) concentrou maior proporção (22,9%). Maiores prevalências associaram-se ao sexo ignorado (RP 1,18; IC95% 1,11; 1,23), raça/cor da pele preta (RP 1,17; IC95% 1,16; 1,17) e indígena (RP 1,16; IC95% 1,10; 1,22), faixa etária ≥80 anos (RP 1,74; IC95% 1,71; 1,75) e óbitos em local ignorado (RP 1,30; IC95% 1,25; 1,34).

**Conclusão:**

Metade dos óbitos apresentou causa básica registrada como código garbage, com maior prevalência entre sexo e local de ocorrência ignorados, idosos e raça/cor da pele preta e indígena. Investigação ativa, redistribuição por algoritmos e integração de bancos de dados mostram-se promissores para qualificar a informação.

**Aspectos éticos**

Esta pesquisa utilizou bancos de dados de domínio público e anonimizados

## Introdução

As estatísticas de mortalidade são fontes valiosas de informação em saúde para a avaliação do estado de saúde das populações [1]. A utilidade desses dados para a gestão pública depende da correta identificação da causa básica do óbito, definida como “a doença ou lesão que iniciou a cadeia de eventos patológicos que conduziram à morte ou as circunstâncias do acidente ou violência que produziram a lesão fatal” [2]. Contudo, a qualidade dessa informação é frequentemente comprometida pela utilização dos códigos garbage ou por causas inespecíficas de óbito, as quais consistem em diagnósticos indefinidos, incompletos ou pouco específicos que impedem a identificação adequada de ações prioritárias em saúde pública [3].

Para qualificar o enfrentamento desse problema, o Global Burden of Disease Study (GBD) propôs uma taxonomia que hierarquiza os códigos garbage em quatro níveis, baseando-se no grau de inespecificidade e no impacto na distorção das estatísticas de mortalidade, sendo o nível 1 de muito alto impacto e o nível 4 de baixo impacto. Essa classificação permite identificar causas que impedem o reconhecimento do grupo da doença até diagnósticos que carecem de maior detalhamento. A adoção dessa tipologia subsidia processos de redistribuição de óbitos e orienta gestores na priorização de intervenções [4].

Embora as estatísticas de mortalidade segundo causa sejam importantes para caracterização do perfil epidemiológico da população e de priorização de ações e políticas de saúde [5], a baixa qualidade das bases de dados limita tais análises, especialmente nos países em desenvolvimento [5-7]. Uma alta proporção de códigos garbage compromete a qualidade da informação sobre causas de morte e tem sido usada como indicador para avaliação da qualidade da assistência, visto que a desigualdade no acesso e na qualidade da atenção médica são relacionados à frequência de óbitos por causas inespecíficas [7,8]. Estima-se que, quando a proporção de códigos garbage ultrapassa 10% do total de mortes, os dados de mortalidade ficam comprometidos, o que dificulta a obtenção de estatísticas confiáveis [9].

No Brasil, proporções de códigos garbage acima de 30% persistem [3,9-11], porém a literatura sobre o tema ainda carece de estudos localizados, o que justifica a contribuição desta análise para o território do estado do Rio de Janeiro.

Os objetivos deste estudo foram descrever a evolução temporal e a distribuição geográfica dos óbitos por códigos garbage, além de analisar a prevalência e as características associadas à ocorrência de códigos garbage como causa básica de óbito no estado do Rio de Janeiro entre 1998 e 2023.

## Métodos

### Delineamento, local, período e população do estudo

Trata-se de estudo transversal com base em dados individuais de todos os óbitos de residentes no estado do Rio de Janeiro registrados no Sistema de Informação sobre Mortalidade (SIM) entre 1º de janeiro de 1998 e 31 de dezembro de 2023. Esse estado compreende 92 municípios distribuídos em nove regiões de saúde com população estimada, em 2022, de 16.054.524 habitantes [12].

Foram realizadas análises descritivas agregadas por causa básica, ano e município, para caracterizar o contexto espacial e temporal dos óbitos, bem como análises ao nível individual para estimar a prevalência e identificar fatores associados ao registro de códigos garbage. 

### Variáveis 

A classificação da variável dependente deste estudo foi definida pela presença de qualquer causa básica de óbito classificada como código *garbage *nos níveis 1 a 4, seguindo a metodologia do GBD. Utilizou-se a causa básica do óbito codificada conforme a 10ª revisão da Classificação Internacional de Doenças (CID-10), incluindo os códigos de quatro dígitos.

A metodologia seguiu a classificação dos códigos garbage proposta pelo GBD de 2017, que categorizou os códigos da CID-10 em quatro níveis de qualidade (Tabela Suplementar 1):

- nível 1 (pior qualidade) – inclui os códigos com sérias implicações para políticas públicas, como septicemia, que podem ser redistribuídos para qualquer grande grupo;
- nível 2 (baixa qualidade) – inclui os códigos com implicações substanciais para causas que podem ser redistribuídas em um ou dois grandes grupos, a exemplo da hipertensão essencial;
- nível 3 (média qualidade) – inclui os códigos com implicações importantes para causas que provavelmente estão no mesmo capítulo da CID-10, como o câncer de localização não especificada; e
- nível 4 (melhor qualidade) – para causas que se referem a uma única doença, mas que poderiam ser mais especificadas, como o AVC não especificado.

Tais códigos são posteriormente realocados para causas básicas prováveis por algoritmos de redistribuição baseados em proporções de registros de alta qualidade da mesma população (15).

As variáveis independentes selecionadas para a análise foram: sexo (masculino, feminino, ignorado), raça/cor da pele (branca, preta, amarela, parda, indígena, ignorado), faixa etária (<1, 1-9, 10-19, 20-39, 40-59, 60-79 e ≥80 anos), local de ocorrência do óbito (hospital, domicílio, via pública, outros estabelecimentos de saúde, outros, ignorado), região de saúde (Baía da Ilha Grande, Baixada Litorânea, Centro-Sul, Médio Paraíba, Metropolitana I, Metropolitana II, Noroeste, Norte e Serrana) e ano do óbito (1998-2023).

Os anos do óbito foram analisados de forma contínua (1998-2023) na análise descritiva e como categóricos para a análise multivariada. Para apresentação na tabela de regressão, foram selecionados: 1998 (início da série histórica com CID-10 performando estabilidade); 2011 (ano central da série, com qualidade intermediária após investimentos nacionais em vigilância); 2021 (momento mais crítico da pandemia de covid-19); e 2023 (último ano disponível).

### Fontes de dados e mensuração

A fonte para obtenção dos dados foi o SIM, disponibilizado pelo Departamento de Informática do Sistema Único de Saúde (DATASUS), cujo banco de dados com informações não identificadas encontra-se publicamente disponível no repositório de dados OpenDATASUS [13].

O processamento dos dados de mortalidade no estado do Rio de Janeiro apresenta fluxo descentralizado. O ciclo inicia-se com a emissão da declaração de óbito por médicos das unidades notificadoras, cabendo às secretarias municipais de Saúde coleta, digitação e processamento dos dados. A codificação das causas e a seleção da causa básica são realizadas por codificadores capacitados vinculados às esferas municipal e estadual, com auxílio do software Seletor de Causa Básica para aplicação das regras da CID-10. O nível estadual monitora qualidade, corrige inconsistências e valida os dados antes do processamento final pelo DATASUS [14].

Para a análise, utilizou-se a base de dados original conforme disponibilizada pelo Ministério da Saúde, sem a exclusão de registros por incompletude de variáveis sociodemográficas. As categorias classificadas como “ignorado” foram mantidas e analisadas como tal, refletindo o cenário real do preenchimento das declarações de óbito no período.

### Métodos estatísticos 

Inicialmente, foram realizadas análises descritivas da distribuição do número total de óbitos (n) e das proporções (%) de códigos garbage para cada categoria das variáveis de estudo. A proporção de códigos garbage total e pelos níveis ao longo dos anos foi apresentada em forma de gráfico.

A distribuição geográfica da proporção de códigos garbage por município foi visualizada em mapas, utilizando a unidade territorial municipal e o método de classificação por intervalos iguais, com a definição de cinco faixas fixas de 10 pontos percentuais cada (20%-30%, 31%-40%, 41%-50%, 51%-60% e >60%). Adicionalmente, os resultados foram estratificados pelas nove regiões de saúde do estado, o que permitiu a comparação do desempenho da qualidade da informação entre tais agregados regionais.

Foi realizado um ranqueamento das cinco causas básicas de óbito mais frequentes em cada um dos quatro níveis de qualidade propostos pelo GBD, utilizando a frequência absoluta (N), a proporção de cada causa no seu nível. As proporções foram calculadas utilizando como denominador o total de óbitos classificados em cada nível do GBD, apresentando-se as cinco causas de maior frequência por nível.

Devido à alta prevalência do evento, a associação entre as variáveis dependentes e independentes foi modelada por regressão de Poisson com variância robusta, abordagem adequada para obtenção da razão de prevalência (RP) ajustada em estudos transversais com desfechos comuns, evitando a superestimação da magnitude da associação decorrente do uso da razão de chances (*odds ratio*, OR).

As associações foram inicialmente investigadas por análises univariadas para a obtenção das RPs brutas. Em seguida, procedeu-se à modelagem multivariável, na qual o modelo final foi ajustado simultaneamente pelas variáveis sexo, raça/cor da pele, faixa etária, local de ocorrência, região de saúde e ano do óbito. Essa abordagem foi adotada por tratar-se de uma série histórica longa, na qual o ajuste se faz necessário para o controle de fatores de confusão decorrentes de mudanças na composição demográfica e espacial da população. Isso garante que as RPs reflitam as associações ajustadas para cada variável analisada.

As estimativas de associação foram expressas como RP, acompanhadas dos respectivos intervalos de confiança de 95% (IC95%). A significância estatística foi avaliada pelo teste de Wald com erro-padrão robusto, adotando-se o nível de significância de 5%.

As análises foram realizadas com auxílio dos programas Stata 14.2 e R Studio 4.0.2.

## Resultados

No período 1998-2023, foram analisados 3.399.380 óbitos de residentes no estado do Rio de Janeiro, sendo 1.510.135 (44,4%) classificados como códigos garbage.

A proporção de óbitos por códigos garbage no estado permaneceu elevada ao longo dos anos (Figura 1A), variando de 48,0% em 1998 para 46,3% em 2023. As menores proporções foram observadas em 2021 (38,1%) e 2020 (38,8%), enquanto as maiores foram em 1998 (48,0%) e 2019 (47,4%). 

**Figura 1:** Evolução temporal (A) e distribuição por níveis (B) da proporção (%) de óbitos por códigos garbage (CG). Estado do Rio de Janeiro, 1998-2023 (n=1.510.135)

Detalhou-se a proporção de óbitos por códigos garbage por nível de qualidade do GBD em relação ao total de óbitos (Figura 1B). O nível 1 abrangeu as causas mais inespecíficas e de pior qualidade. Esse nível concentrou a maior proporção, mantendo-se em 22,9% no início (1998) e no final da série (2023), com ponto mais baixo em 2016 (18,8%). Seguiu-se o nível 4, com variação de 14,0% em 1998 para 13,4% em 2023, com o mínimo em 9,6% em 2021. O nível 2 variou de 6,8% em 1998 para 7,3% em 2023, e o nível 3 de 4,3% em 1998 para 3,1% em 2023, tendo seu mínimo em 2,4% em 2021. Observou-se queda nas proporções de códigos garbage em todos os níveis em 2020 e 2021. Esse declínio foi seguido por importante aumento em 2022 e 2023, com as proporções retornando a patamares próximos à pré-pandemia da covid-19.

Foi apresentado o ranqueamento das cinco causas de códigos garbage mais frequentes em cada nível de qualidade do GBD (Tabela 1). No nível 1, a causa R99 (outras causas mal definidas e as não especificadas de mortalidade) foi a mais frequente e correspondeu a 32,5% dos óbitos nesse nível (n=232.787). No nível 2, a hipertensão essencial (primária) (I10) respondeu pela maior proporção dos óbitos, 39,4% (n=88.799). O nível 3 destacou outros transtornos pulmonares (J98.4, 17,4%) e neoplasia maligna sem especificação de localização (C80, 14,3%). No nível 4, a pneumonia não especificada (J18.9, 33,2%) e o acidente vascular cerebral não especificado (I64, 26,7%) foram as causas mais comuns.

**Tabela 1:** Cinco causas básicas de óbito mais frequentes de cada nível de códigos garbage segundo Global Burden of Disease Study (GBD), frequência absoluta e proporção em cada nível. Estado do Rio de Janeiro, 1998-2023 (n=1.510.135)

Código CID-10^a^	Descrição da causa básica	n (%)^b^
**Nível 1 **– **Pior qualidade**		
R99	Outras causas mal definidas e as não especificadas de mortalidade	232.787 (32,5)
E149	Diabetes mellitus não especificado	87.567 (12,2)
A419	Septicemia não especificada	77.872 (10,9)
I500	Insuficiência cardíaca congestiva	36.632 (5,1)
I509	Insuficiência cardíaca não especificada	27.350 (3,8)
**Nível 2 **– **Qualidade baixa**		
I10	Hipertensão essencial (primária)	88.799 (39,4)
Y349	Eventos não especificados e intenção não determinada	31.023 (13,8)
R092	Parada respiratória	19.104 (8,5)
J81	Edema pulmonar	14.281 (6,3)
K920	Hematêmese	11.862 (5,3)
**Nível 3 **– **Qualidade média**		
J984	Outros transtornos pulmonares	20.360 (17,4)
C80	Neoplasia maligna sem especificação de localização	16.706 (14,3)
I499	Arritmia cardíaca não especificada	9.653 (8,2)
J988	Outros transtornos respiratórios especificados	9.245 (7,9)
C260	Neoplasia maligna do trato intestinal, parte não especificada	7.928 (6,8)
**Nível 4 **– **Melhor qualidade**		
J189	Pneumonia não especificada	150.091 (33,2)
I64	Acidente vascular cerebral, não especificado como hemorrágico ou isquêmico	120.713 (26,7)
I678	Outras doenças cerebrovasculares especificadas	47.548 (10,5)
I694	Sequelas de acidente vascular cerebral, não especificado como hemorrágico ou isquêmico	28.541 (6,3)
I420	Cardiomiopatia dilatada	24.126 (5,3)

^a^Classificação Internacional de Doenças – 10ª revisão (CID-10); ^b^As proporções foram calculadas sobre o total de óbitos de cada nível do GBD, considerando-se apenas as cinco causas de maior frequência por nível.

A distribuição geográfica da proporção de óbitos por códigos garbage por município foi ilustrada para 1998, 2011, 2021 e 2023 (Figura 2). Em 1998 (Figura 2A), as maiores proporções estavam em municípios do Noroeste fluminense, como Varre-Sai (68,4%), e na Região Metropolitana I, em Queimados (68,1%). Observou-se variação geral de tons mais escuros (altas proporções) em grande parte do estado. 

**Figura 2:** Proporção de óbitos por códigos garbage por município: (A) 1998, (B) 2011, (C) 2021 e (D) 2023. Estado do Rio de Janeiro (n=1.510.135)

Em 2011 (Figura 2B), a maioria dos municípios apresentou redução nas proporções de óbitos por códigos garbage, visível pelo clareamento nos mapas. Regiões como Metropolitana I, Metropolitana II e Centro-Sul exibiram as menores proporções, com Areal (34,44%) na Centro-Sul e Niterói (37,5%) na Metropolitana II entre os municípios com menores proporções. Municípios do Noroeste, como Varre-Sai (47,5%), ainda mantinham proporções elevadas. 

Em 2021 (Figura 2C), a proporção de óbitos por códigos garbage apresentou redução observada em grande parte do estado em comparação aos anos anteriores, refletindo o período da pandemia de covid-19. Municípios da Região Serrana, como Petrópolis (27,9%), São José do Vale do Rio Preto (29,5%) e São Sebastião do Alto (38,0%), apresentaram as menores proporções. 

Em 2023 (Figura 2D), a proporção de óbitos por códigos *garbage *aumentou de forma importante em diversas regiões, com o mapa escurecendo novamente em comparação a 2021. As maiores proporções foram registradas em municípios da Baixada Litorânea, como Rio das Ostras (59,48%), e da Região Metropolitana I, como Belford Roxo (50,3%).

Foram apresentadas RPs ajustadas para a proporção de óbitos por códigos garbage, com seus respectivos IC95% (Tabela 2). Todas as categorias apresentaram significância estatística (p-valor<0,001). A prevalência de óbitos por códigos garbage foi 6,0% superior no sexo feminino em comparação ao masculino (RP 1,06; IC95% 1,06; 1,06). Para a raça/cor da pele, as maiores RPs ajustadas foram observadas nas populações preta (RP 1,17; IC95% 1,16; 1,17) e indígena (RP 1,16; IC95% 1,10; 1,22) em comparação à população branca. 

**Tabela 2:** Razão de prevalência (RP) bruta e ajustada e intervalo de confiança de 95% (IC95%) de óbitos por códigos garbage segundo características sociodemográficas. Estado do Rio de Janeiro, 1998-2023 (n=3.399.380)

Variáveis	Total de óbitos	Óbitos classificados como códigos garbage – n (%)	RP bruta (IC95%)	p-valor	RP ajustada (IC95%)^a,d^	p-valor
Sexo				<0,001		<0,001
Masculino	1.837.839	770.190 (41,9)	1		1	
Feminino	1.558.618	738.311 (47,4)	1,13 (1,12; 1,13)		1,06 (1,06; 1,06)	
Ignorado	2.923	1.634 (55,9)	1,33 (1,29; 1,38)		1,18 (1,11; 1,23)	
**Raça/cor da pele**				<0,001		<0,001
Branca	1.827.802	789.877 (43,2)	1		1	
Preta	489.258	233.242 (47,7)	1,10 (1,10; 1,11)		1,17 (1,16; 1,17)	
Amarela	5.822	2.671 (45,8)	1,06 (1,03; 1,09)		1,06 (1,03; 1,09)	
Parda	961.009	428.903 (44,6)	1,03 (1,03; 1,04)		1,13 (1,12; 1,13)	
Indígena	1.713	834 (48,7)	1,13 (1,07; 1,18)		1,16 (1,10; 1,22)	
Ignorado	113.776	54.608 (48,0)	1,11 (1,10; 1,12)		1,15 (1,13; 1,15)	
**Faixa etária **(anos)^b^				<0,001		<0,001
<1	88.298	14.671 (16,6)	0,56 (0,55; 0,57)		0,52 (0,51; 0,53)	
1- 9	21.756	9.005 (41,4)	1,40 (1,37; 1,43)		1,30 (1,27; 1,32)	
10-19	60.090	17.799 (29,6)	1		1	
20-39	292.051	91.843 (31,4)	1,06 (1,05; 1,08)		1,04 (1,02; 1,05)	
40-59	679.181	272.006 (40,0)	1,35 (1,34; 1,37)		1,28 (1,26; 1,29)	
60-79	1.341.140	617.182 (46,0)	1,55 (1,53; 1,57)		1,49 (1,46; 1,50)	
≥80	916.864	487.629 (53,2)	1,80 (1,77; 1,82)		1,74 (1,71; 1,75)	
**Local de ocorrência**				<0,001		<0,001
Hospital	2.441.787	1.062.323 (43,5)	1		1	
Ignorado	3.269	1.759 (53,8)	1,24 (1,20; 1,28)		1,30 (1,25; 1,34)	
Outros estabelecimentos de saúde	255.956	139.001 (54,3)	1,25 (1,24; 1,25)		1,26 (1,25; 1,26)	
Domicílio	468.159	227.807 (48,7)	1,12 (1,11; 1,12)		1,08 (1,07; 1,08)	
Via pública	129.966	37.181 (28,6)	0,66 (0,65; 0,66)		0,74 (0,72; 0,74)	
Outros	100.243	42.064 (42,0)	0,96 (0,96; 0,97)		1,01 (1,00; 1,02)	
**Região de saúde**				<0,001		<0,001
Baía da Ilha Grande	36.804	14.269 (38,8)	0,86 (0,85; 0,87)		0,92 (0,90; 0,92)	
Baixada Litorânea	137.815	62.673 (45,5)	1,00 (1,00; 1,01)		1,04 (1,03; 1,04)	
Centro-Sul	70.304	30.957 (44,0)	0,97 (0,96; 0,98)		0,98 (0,97; 0,99)	
Médio Paraíba	168.556	66.614 (39,5)	0,87 (0,87; 0,88)		0,90 (0,89; 0,90)	
Metropolitana I	2.111.347	955.390 (45,2)	1		1	
Metropolitana II	403.987	175.141 (43,3)	0,96 (0,95; 0,96)		0,97 (0,97; 0,98)	
Noroeste	72.405	31.117 (43,0)	0,95 (0,94; 0,96)		0,95 (0,94; 0,96)	
Norte	159.752	72.305 (45,3)	1,00 (0,99; 1,01)		1,04 (1,03; 1,04)	
Serrana	195.953	79.614 (40,6)	0,90 (0,89; 0,90)		0,92 (0,91; 0,92)	
Ignorado	42.457	22.055 (51,9)	1,15 (1,14; 1,16)		1,08 (1,07; 1,09)	
**Ano do ^óbitoc^**				<0,001		<0,001
1998	115.382	55.383 (48,1)	1		1	
2011	127.095	55.718 (43,8)	0,91 (0,90; 0,92)		0,85 (0,84; 0,86)	
2021	189.232	72.165 (38,1)	0,79 (0,79; 0,80)		0,71 (0,70; 0,71)	
2023	145.148	67.133 (46,3)	0,96 (0,96; 0,97)		0,85 (0,84; 0,86)	

^a^Modelo ajustado por todas as variáveis independentes; ^b^A faixa etária 10-19 anos foi selecionada como categoria de referência por apresentar a segunda menor prevalência de códigos garbage, imediatamente após os menores de 1 ano, cujos óbitos são obrigatoriamente investigados; ^c^Os anos do óbito foram analisados sem estratificação temporal, no entanto, para apresentação, foram escolhidos anos específicos que correspondem a: 1998 (início da série histórica com CID-10 estável); 2011 (ano central da série história estudada, com qualidade intermediária após investimentos nacionais em vigilância); 2021 (momento crítico da pandemia de covid-19); e 2023 (último ano disponível priorizando clareza visual em momentos-chave); ^d^A sobreposição dos limites do IC95% à estimativa pontual deve-se à elevada precisão estatística decorrente da magnitude da amostra, mantendo-se a padronização de duas casas decimais.

A prevalência de óbitos por códigos garbage foi menor em crianças menores de 1 ano (RP 0,52; IC95% 0,51; 0,53) e aumentou com o envelhecimento, sendo 74,0% superior para 80 anos ou mais em comparação à faixa de 10-19 anos (RP 1,74; IC95% 1,71; 1,75). 

A prevalência foi maior nas categorias ignorado (RP 1,30; IC95% 1,25; 1,34) e outros estabelecimentos de saúde (RP 1,26; IC95% 1,25; 1,26) em comparação a óbitos ocorridos em hospitais e 26,0% menor para óbitos em Via Pública (RP 0,74; IC95% 0,72; 0,74). As regiões de saúde Baixada Litorânea (RP 1,04; IC95% 1,03; 1,04) e Norte (RP 1,04; IC95% 1,03;1,04) apresentaram RPs maiores que a Metropolitana I, enquanto Médio Paraíba (RP 0,90; IC95% 0,89; 0,90), Serrana (RP 0,92; IC95% 0,91; 0,92) e Baía da Ilha Grande (RP 0,92; IC95% 0,90; 0,92) tiveram as menores. 

Observou-se redução na prevalência de óbitos por códigos garbage ao longo da série histórica. Comparado a 1998, a RP foi menor em todos os anos subsequentemente avaliados, atingindo o ponto mais baixo em 2021 (RP 0,71; IC95% 0,70; 0,71). Em 2023, a prevalência ajustada permaneceu 15,0% abaixo daquela observada em 1998 (RP 0,85; IC95% 0,84; 0,86).

## Discussão

Os resultados deste estudo destacaram-se pela magnitude e persistência dos óbitos classificados como códigos garbage, o que indicou desafios históricos na qualificação da informação sobre mortalidade no estado do Rio de Janeiro. Apesar dos avanços, sua ocorrência permaneceu sistematicamente acima dos parâmetros considerados aceitáveis para garantir estatísticas de mortalidade confiáveis [9] e foram corroborados por achados em outros territórios brasileiros, que mostraram que 19 estados apresentaram a proporção de códigos garbage superior a 30% em 2016 e nenhum inferior a 10% [3,6,9,11,15]. 

A redução na proporção de códigos garbage entre 2020 e 2021 pode ser atribuída ao maior rigor na investigação de óbitos, à especificidade da covid-19 como causa básica de morte bem-definida e à mobilização dos sistemas de vigilância no estado do Rio de Janeiro, hipótese que diverge de outros estudos realizados na pandemia, que observaram aumento nesse período. Todavia, é necessário cautela ao interpretar essa redução como melhora estrutural na qualidade, visto que é possível que tenha ocorrido priorização na escolha da causa covid-19 em detrimento de causas crônicas subjacentes [10,16,17].

O subsequente aumento das proporções de códigos garbage, em 2022 e 2023, sugeriu que a melhora na qualidade dos dados não se sustentou, com os níveis de inespecificidade retornando a patamares elevados no período pós-pandemia da covid-19.

A concentração de códigos garbage no nível 1 representou o comprometimento mais severo na qualidade da informação. A predominância de códigos como R99 (outras causas mal definidas) limitou o uso das estatísticas, pois a ausência total de detalhamento etiológico impossibilitou a identificação da real causa de óbito e a definição de prioridades no planejamento de ações de saúde pública. Isso reforçou a necessidade de investimentos em investigação de óbitos e melhor capacitação dos profissionais responsáveis pelo preenchimento das declarações de óbito [4,18]. 

Os códigos classificados nos níveis 2 e 3, como a hipertensão essencial e a neoplasia maligna sem localização anatômica definida, respectivamente, guardaram potencial de qualificação superior aos códigos de nível 1, pois esclareceram, ao menos, o sistema orgânico ou o grupo de causas envolvido. No entanto, ainda denotaram uma oportunidade perdida para aprofundar o conhecimento sobre a morbimortalidade por doenças crônicas não transmissíveis no estado do Rio de Janeiro [19,20].

As disparidades na proporção de códigos garbage entre as regiões de saúde e os municípios refletiram a heterogeneidade das condições de saúde e da capacidade de registro no estado. Estes achados indicaram possíveis desigualdades na qualidade da informação, na capacidade local de investigação de óbitos e, em última instância, na oferta e no acesso aos serviços de saúde [5].

Os resultados reforçaram o papel dos códigos garbage como indicador indireto de fragilidades na atenção à saúde, especialmente em municípios do interior ou em áreas metropolitanas vulneráveis, onde concentrações elevadas podem sinalizar problemas de cobertura e de qualidade assistencial e capacitação técnica [16]. Municípios com menores proporções de códigos garbage podem indicar melhores práticas locais de preenchimento ou investigação de óbitos, enquanto aqueles com maiores proporções sugerem a necessidade de intervenções direcionadas [21], especialmente onde há prevalência superior a 50% ao longo de 25 anos.

As diferenças observadas nas prevalências de códigos garbage estratificadas por variáveis sociodemográficas sublinharam a influência dos determinantes sociais da saúde e das iniquidades no acesso e na qualidade da atenção entre os subgrupos populacionais [7]. As maiores RPs associadas à raça/cor da pele preta e indígena reforçaram a interface entre qualidade da informação e iniquidades em saúde. Isso sugeriu barreiras de acesso à investigação diagnóstica, que podem se estender ao processo de certificação do óbito, resultando em dados menos precisos [22].

Em relação ao sexo, observou-se maior prevalência em óbitos do sexo feminino. Isso pode estar associado à maior ocorrência de óbitos por causas externas em homens, que são, em geral, certificados por legistas após necropsias, o que levaria ao diagnóstico específico das causas da morte para o sexo masculino [17].

A menor RP em crianças menores de 1 ano sugeriu maior rigor na investigação de óbitos infantis, enquanto o aumento progressivo em faixas etárias avançadas apontou para a complexidade diagnóstica e a prevalência de comorbidades em idosos como fatores que contribuem para a inespecificidade das causas de morte nesse grupo. Tal fato poderia explicar, também, a maior frequência entre mulheres, dado à maior expectativa de vida em comparação aos homens [17,23].

A associação de maiores prevalências de códigos garbage em óbitos ocorridos em domicílio, via pública ou em outros estabelecimentos de saúde, em comparação a óbitos hospitalares, é um padrão consistente com a literatura. Óbitos fora do ambiente hospitalar geralmente carecem de informações clínicas mais detalhadas, o que torna a determinação da causa básica mais desafiadora. Já a menor proporção em vias públicas pode estar relacionada à melhor investigação de óbitos por causas externas, visto que estes são obrigatoriamente investigados por médicos legistas a partir de necropsias, o que culmina em causas básicas específicas [3,8,9]. 

Embora a proporção de códigos garbage tenha se mantido elevada ao longo do tempo, a análise das RPs revelou uma trajetória de melhora gradual na qualidade da informação ao longo da série histórica. Essa tendência de declínio, ainda que insuficiente, sugeriu que investimentos em vigilância e qualificação do preenchimento das declarações de óbito podem ter tido efeito positivo ao longo dos anos. 

Os achados reforçaram a necessidade de investimentos contínuos em qualificação dos profissionais de saúde que atestam, certificam e investigam as causas de óbito, especialmente médicos e equipes de vigilância. Destacou-se a urgência de fortalecer a rede de vigilância de óbitos no estado, com especial atenção aos municípios com maiores proporções de causas de óbito inespecíficas. 

A investigação dos óbitos é estratégia indispensável para qualificar a informação e deve integrar o processo de trabalho das equipes de vigilância locais. Ao investigar ativamente óbitos por causas inespecíficas, os serviços de vigilância corrigem distorções nos dados e identificam fragilidades na assistência e barreiras de acesso, o que permite intervenções direcionadas aos territórios e aos grupos populacionais mais afetados [24-27]. 

Outra estratégia é a reclassificação de óbitos por códigos *garbage *utilizando algoritmos padronizados de redistribuição. Estudos que aplicaram essa correção demonstraram que o perfil de mortalidade é substancialmente alterado, revelando o verdadeiro peso de causas específicas [20,28,29]. 

A integração entre sistemas de informação por técnicas de relacionamento de bancos de dados apresenta-se como ferramenta para qualificar a investigação de óbitos por causas inespecíficas. O relacionamento de registros do SIM com bases assistenciais permite recuperar trajetórias de cuidado e dados clínicos ausentes na declaração de óbito. Essa estratégia, ao enriquecer o contexto do óbito, pode subsidiar a reclassificação desses códigos para causas específicas, o que confere maior fidedignidade às estatísticas de mortalidade [30]. 

Entre as limitações, a utilização de dados secundários do SIM é sujeita a vieses de informação relacionados a sub-registro, bem como a incompletude e inconsistências no preenchimento das declarações de óbito. Embora a metodologia do GBD seja amplamente aceita, pode não captar particularidades do contexto brasileiro. Por tratar-se de estudo transversal, as associações não permitiram inferências causais. A presença de informação ignorada pode ter influenciado as estimativas das RPs ajustadas. Contudo, o grande volume de dados e o uso de metodologia validada fortaleceram a robustez dos resultados.

Em conclusão, o estado do Rio de Janeiro enfrenta desafios na qualidade dos dados de mortalidade, caracterizados por elevada magnitude e persistência dos códigos garbage, com marcada heterogeneidade temporal e territorial, associada a desigualdades sociodemográficas. Os resultados deste estudo permitem orientar a priorização de municípios, regiões e subgrupos mais vulneráveis para implementar estratégias contínuas de aprimoramento do preenchimento das declarações de óbito e da investigação das causas de morte, visando fortalecer as estatísticas vitais para o planejamento e a avaliação das políticas de saúde.

## Data Availability

Os bancos de dados derivados e os códigos de análise estão disponíveis mediante solicitação à autora.
